# Microglia matters: visualizing the immune battle in Parkinson’s disease

**DOI:** 10.1172/JCI192919

**Published:** 2025-06-16

**Authors:** So Jeong Lee, Changning Wang, Jacob Hooker

**Affiliations:** Athinoula A. Martinos Center for Biomedical Imaging, Department of Radiology, Massachusetts General Hospital, Harvard Medical School, Charlestown, Massachusetts, USA.

## Abstract

Microglia play critical roles in immune defense within the central nervous system (CNS), and microglia-mediated immune changes in the brain are observed in various neurodegenerative diseases, including Parkinson’s disease (PD). While PET imaging with a range of radiolabeled ligands has been invaluable for visualizing and quantifying neuroimmune changes in the brains of patients with PD, no PET ligands currently exist that are specific to microglia. In this issue of the *JCI*, Mills et al. used the PET radioligand [¹¹C]CPPC to image colony stimulating factor 1 receptor (CSF1R), revealing a connection between increased CSF1R expression and microglia-mediated brain immune changes in patients with PD. The study demonstrated that elevated CSF1R expression colocalized with a microglial-specific marker in brain regions vulnerable to PD. Moreover, quantifying CSF1R density with [¹¹C]CPPC-PET imaging in living brains may provide an indicator of motor and cognitive impairments in the early stages of PD. These findings underscore the potential of CSF1R-PET imaging as a microglial-sensitive biomarker of brain immune function in PD.

## Microglial-mediated neuroimmune response as a biomarker in PD

Disruption of the CNS immune system, including microglial activation of neurotoxic astrocytes, is an early pathological event linked to α-synuclein (α-Syn) misfolding and neurodegeneration in Parkinson’s disease (PD) (1–3). Once the balance between innate immunity and α-Syn propagation that activates microglia is disturbed, a cycle of immune cell dysfunction and neurodegeneration ensues, promoting disease progression (4–6). Thus, identifying a specific, noninvasive biomarker to assess microglial activation is a crucial step in developing targeted PD therapies.

For over 20 years, PET ligands targeting the 18 kDa translocator protein (TSPO) have been widely used to assess CNS immune activation in neurodegenerative diseases like PD (7, 8). TSPO radiotracers have provided valuable insights into neuroinflammation, aligning with pathological findings and advancing our understanding of disease heterogeneity and progression. However, interpreting TSPO-related biological mechanisms is complex because TSPO expression is not specific to a single cell type (9, 10), and TSPO reflects various functional changes such as steroidogenesis, cytokine release, and reactive oxygen generation (11). These factors complicate the interpretation of TSPO-PET imaging, particularly in therapeutic contexts (12). As a result, there is growing interest in developing new, quantitative, noninvasive imaging tools that can specifically measure microglial activation and proliferation, providing a novel biomarker for early-stage PD and its severity.

## Elevated CSF1R expression and microglial activation in PD

Colony-stimulating factor 1 receptor (CSF1R) is a tyrosine kinase primarily expressed by microglia in the brain, with low expression in other cell types such as neurons and astrocytes (13). It regulates microglial development, survival, and function, playing a pivotal role in neuroinflammation (14–16). While CSF1R inhibition has been explored for treating inflammatory and neuroinflammatory disorders (17), its overexpression in the postmortem brain of individuals with PD had not been fully established. In this issue of the *JCI*, Mills and authors used IHC in the postmortem brain to demonstrate colocalization of CSF1R with the microglial marker IBA1 (18). They found elevated CSF1R immunoreactivity in multiple PD brain regions, with the most prominent increases (around 60-fold) in the midbrain, compared with controls without neurodegenerative pathology. These findings confirm that CSF1R was overexpressed in established PD and that it colocalized with microglia. While further studies are necessary to assess CSF1R expression in other immune cells, such as macrophages, these results suggest that CSF1R is highly overexpressed in patients with PD. Additionally, autoradiography using the tritiated CSF1R radioligand [^3^H]JHU11761 revealed CSF1R density in key regions, such as the inferior parietal cortex (IPC), caudate nucleus (CN), midbrain (MB), and basal ganglia (BG), in both grey matter (GM) and white matter (WM). The most notable binding differences were observed in IPC, with a 4-fold increase in GM and a 9-fold increase in WM, with no sex differences. Further equilibrium binding studies confirmed elevated CSF1R sites in IPC, CN, and MB in both WM and GM, with increased radiotracer binding in GM subregions of subjects with PD, suggesting heightened CSF1R-related inflammation.

## Measuring neuroimmune changes in early PD pathology

Given the need for in vivo assessment of microglial density and proliferation in the early stages of neurodegeneration in PD, CSF1R has been targeted as a microglial-sensitive marker for PET imaging. Researchers at Johns Hopkins University developed [^11^C]CPPC as a CSF1R-specific PET radiotracer, which has shown fast kinetics and low off-target binding in mouse neuroinflammation models and nonhuman primates (13). It also demonstrated regional distribution corresponding to CSF1R-enriched regions in healthy humans (19, 20).

Mills et al. (18) studied [^11^C]CPPC binding in a cohort of individuals with early PD (i.e., mild to moderate motor disability and cognitive impairment) and age-matched individuals in a healthy control group to explore the relationship between PD severity and tissue-specific [^11^C]CPPC binding. The regional total volume of distribution (V_T_), calculated from kinetic modeling of in vivo PET imaging, measured the ratio of the [^11^C]CPPC concentration in specific tissues relative to the plasma. Mills et al. compared the mean [^11^C]CPPC V_T_ across multiple brain regions in healthy controls and in PD groups with mild or moderate motor disability. They found differences in several brain regions, with the striatum showing the only statistically significant difference (*P* < 0.004). The differences were mainly between the moderate motor disability group and both the mild motor disability and control groups. No differences were found between healthy controls and mild motor disability groups, although a positive correlation between V_T_ and motor disability was observed in several regions. Higher motor disability was associated with greater [^11^C]CPPC binding, with only the brainstem (*r* = 0.78, *P* = 0.003) and temporal cortex (*r* = 0.78, *P* = 0.003) showing statistically significant correlations. No correlations were found between regional [^11^C]CPPC V_T_ and clinician-rated motor scores (Movement Disorder Society-Unified Parkinson’s Disease Rating Scale Part II and III), though a weak, nonsignificant correlation with Part III was observed in regions such as the striatum and pallidum, known to be involved in early PD pathophysiology. Mills et al. also found no differences in [^11^C]CPPC V_T_ between participants with PD with mild cognitive impairment, those without cognitive impairment, and those in the healthy control group. While there was a moderate, nonsignificant correlation between regional [^11^C]CPPC V_T_ and global cognitive function (MoCA), a tendency was observed where worse phonemic verbal fluency was associated with higher [^11^C]CPPC V_T_ in multiple brain regions (18).

## Perspectives and future implications

The findings by Mills et al. (18) open up new avenues for using CSF1R-targeted PET imaging with [^11^C]CPPC to detect early brain immune changes in PD, particularly in patients with moderate disease severity. However, several critical questions remain to be addressed. First, the relatively small variance in [^11^C]CPPC binding observed in both age-matched healthy controls and the least-affected PD group — without outliers indicating natural variability — suggests that there may not be substantial genetic variation in binding affinity to CSF1R. This observation contrasts with the *TSPO* gene, where a single nucleotide polymorphism (rs6971 SNP) can substantially affect the binding of many ligands (21, 22). However, the study was preliminary and not powered to assess early-stage PD. Therefore, this finding underscores the need for further research to evaluate [^11^C]CPPC’s sensitivity in detecting microglial activation, particularly in the earliest stages of the disease. Additionally, the small sample size and potential heterogeneity in the study cohorts point to the importance of larger, longitudinal studies to assess how CSF1R changes over time in PD. Second, while CSF1R overexpression in PD is largely associated with microglia and neuroinflammation, it is also expressed in macrophages (23). Evidence suggests that macrophage infiltration into the CNS can preceded overt microglial changes. This raises the possibility that [^11^C]CPPC-PET might not exclusively reflect microglial activity and could be confounded by macrophage involvement (24, 25). As such, further validation studies are necessary to determine whether [^11^C]CPPC-PET can reliably serve as a microglia-specific marker in PD across different stages.

Looking ahead, CSF1R-targeted PET imaging holds promise as a powerful tool for the early detection of microgliosis in PD. If validated, it could provide critical insights into disease progression and serve as a noninvasive biomarker for clinical trials focused on neuroimmune modulation. Further research into its sensitivity, specificity, its ability to detect longitudinal changes, and its relationship to both microglial and macrophage activity will be essential for translating this technique into clinical practice and improving patient care in PD.

## Version 1 06/16/2025

Electronic publication
